# Benchmarking of robotic and laparoscopic spleen-preserving distal pancreatectomy by using two different methods

**DOI:** 10.1093/bjs/znac352

**Published:** 2022-11-02

**Authors:** Tess M E van Ramshorst, Alessandro Giani, Michele Mazzola, Safi Dokmak, Fadhel Samir Ftériche, Alessandro Esposito, Matteo de Pastena, Sanne Lof, Bjørn Edwin, Mushegh Sahakyan, Ugo Boggi, Emanuele Federico Kauffman, Jean Michel Fabre, Regis Francois Souche, Alessandro Zerbi, Giovanni Butturini, Quintus Molenaar, Bilal Al-Sarireh, Marco V Marino, Tobias Keck, Steven A White, Riccardo Casadei, Fernando Burdio, Bergthor Björnsson, Zahir Soonawalla, Bas Groot Koerkamp, Giuseppe Kito Fusai, Patrick Pessaux, Asif Jah, Andrea Pietrabissa, Thilo Hackert, Mathieu D’Hondt, Elizabeth Pando, Marc G Besselink, Giovanni Ferrari, Mohammad Abu Hilal, for the European Consortium on Minimally Invasive Pancreatic Surgery

**Affiliations:** Department of General Surgery, Istituto Ospedaliero Fondazione Poliambulanza, Brescia, Italy; Amsterdamum UMC, location University of Amsterdam, Department of Surgery, Amsterdam, the Netherlands; Cancer Centre Amsterdam, Amsterdam, the Netherlands; Division of Minimally Invasive Surgical Oncology, ASST Grande Ospedale Metropolitano Niguarda, Milan, Italy; Division of Minimally Invasive Surgical Oncology, ASST Grande Ospedale Metropolitano Niguarda, Milan, Italy; Department of Hepatopancreatobiliary Surgery and Liver Transplantation, Beaujon Hospital, Clichy, France; Department of Hepatopancreatobiliary Surgery and Liver Transplantation, Beaujon Hospital, Clichy, France; Department of Surgery, Pancreas Institute, Verona University Hospital, Verona, Italy; Department of Surgery, Pancreas Institute, Verona University Hospital, Verona, Italy; Amsterdamum UMC, location University of Amsterdam, Department of Surgery, Amsterdam, the Netherlands; Cancer Centre Amsterdam, Amsterdam, the Netherlands; Intervention Centre and Department of Hepatopancreatobiliary Surgery, Oslo University Hospital, also Institute of Medicine, University of Oslo, Oslo, Norway; Intervention Centre, Oslo University Hospital, Oslo, Norway; Department of Surgery, University Hospital of Pisa, Pisa, Italy; Department of Surgery, University Hospital of Pisa, Pisa, Italy; Department of Surgery, Saint-Éloi Hospital, Montpellier, France; Department of Surgery, Saint-Éloi Hospital, Montpellier, France; Department of Biomedical Sciences, Humanitas University, Pieve Emanuele, Italy, and IRCCS Humanitas Research Hospital, Rozzano, Italy; Department of Hepatopancreatobiliary Surgery, Pederzoli Hospital, Peschiera del Garda, Italy; Department of Surgery, Regional Academic Cancer Centre Utrecht, UMC Utrecht Cancer Centre and St Antonius Hospital Nieuwegein, University Medical Centre Utrecht, Utrecht, the Netherlands; Department of Surgery, Morriston Hospital, Swansea, UK; General and Emergency Surgery Department, Azienda Ospedaliera Ospedali Riuniti Villa Sofia-Cervello, Palermo, Italy; General Surgery Department, Istituto Villa Salus, Siracusa, Italy; Department of Surgery, University Medical Centre Schleswig-Holstein, Campus Lübeck, Lübeck, Germany; Department of Surgery, Freeman Hospital, Newcastle upon Tyne, Newcastle, UK; Department of Surgery, Sant’Orsola Malphigi Hospital, Bologna, Italy; Department of Surgery, University Hospital del Mar, Barcelona, Spain; Department of Surgery in Linköping and Department of Biomedical and Clinical Sciences, Linköping University, Linköping, Sweden; Department of Surgery, Oxford University Hospital, Oxford, UK; Department of Surgery, Erasmus Medical Centre, Rotterdam, theNetherlands; Hepatopancreatobiliary and Liver Transplant Unit, Royal Free London, London, UK; Division of Hepato-Biliary and Pancreatic Surgery, Nouvel Hôpital Civil, University Hospital, Institut Hospitalo-Universitaire de Strasbourg, Strasbourg, France; Department of Surgery, Cambridge University Hospitals NHS Foundation Trust, Cambridge, UK; Department of Surgery, Fondazione IRCCS Policlinico San Matteo, Pavia, Italy; Department of Surgery, Heidelberg University Hospital, Heidelberg, Germany; Department of Digestive and Hepatobiliary/Pancreatic Surgery, Groeninge Hospital, Kortrijk, Belgium; Department of Surgery, Vall d’Hebron University Hospital, Barcelona, Spain; Amsterdamum UMC, location University of Amsterdam, Department of Surgery, Amsterdam, the Netherlands; Cancer Centre Amsterdam, Amsterdam, the Netherlands; Division of Minimally Invasive Surgical Oncology, ASST Grande Ospedale Metropolitano Niguarda, Milan, Italy; Department of General Surgery, Istituto Ospedaliero Fondazione Poliambulanza, Brescia, Italy; Department of Surgery, University Hospital Southampton NHS Foundation Trust, Southampton, UK

## Abstract

**Background:**

Benchmarking is an important tool for quality comparison and improvement. However, no benchmark values are available for minimally invasive spleen-preserving distal pancreatectomy, either laparoscopically or robotically assisted. The aim of this study was to establish benchmarks for these techniques using two different methods.

**Methods:**

Data from patients undergoing laparoscopically or robotically assisted spleen-preserving distal pancreatectomy were extracted from a multicentre database (2006–2019). Benchmarks for 10 outcomes were calculated using the Achievable Benchmark of Care (ABC) and best-patient-in-best-centre methods.

**Results:**

Overall, 951 laparoscopically assisted (77.3 per cent) and 279 robotically assisted (22.7 per cent) procedures were included. Using the ABC method, the benchmarks for laparoscopically assisted and robotically assisted spleen-preserving distal pancreatectomy respectively were: 150 and 207 min for duration of operation, 55 and 100 ml for blood loss, 3.5 and 1.7 per cent for conversion, 0 and 1.7 per cent for failure to preserve the spleen, 27.3 and 34.0 per cent for overall morbidity, 5.1 and 3.3 per cent for major morbidity, 3.6 and 7.1 per cent for pancreatic fistula grade B/C, 5 and 6 days for duration of hospital stay, 2.9 and 5.4 per cent for readmissions, and 0 and 0 per cent for 90-day mortality. Best-patient-in-best-centre methodology revealed milder benchmark cut-offs for laparoscopically and robotically assisted procedures, with operating times of 254 and 262.5 min, blood loss of 150 and 195 ml, conversion rates of 5.8 and 8.2 per cent, rates of failure to salvage spleen of 29.9 and 27.3 per cent, overall morbidity rates of 62.7 and 55.7 per cent, major morbidity rates of 20.4 and 14 per cent, POPF B/C rates of 23.8 and 24.2 per cent, duration of hospital stay of 8 and 8 days, readmission rates of 20 and 15.1 per cent, and 90-day mortality rates of 0 and 0 per cent respectively.

**Conclusion:**

Two benchmark methods for minimally invasive distal pancreatectomy produced different values, and should be interpreted and applied differently.

## Teaser

This study established benchmark values for laparoscopically and robotically assisted spleen-preserving distal pancreatectomy in both unselected and low-risk patients using two validated methodologies. The benchmark values require different interpretation and application based on the purpose of benchmarking and the patient cohort, and can be used for in-hospital and interhospital comparison and improvement purposes.

## Introduction

Benchmarking is a process in which the performance of best-in-class performers is measured to establish reference values to enable comparison of outcomes against those of the best in the industry^1^. Lately, there has been growing interest in this concept from the surgical field, considering that benchmarks can encourage surgeons to reach the highest possible level of clinical quality and not just perform to the average^2^.

Accordingly, benchmarks have already been established for breast^3^, liver^4–8^, oesophageal^9^, and pancreatic^10–13^ surgery. For pancreatic surgery, benchmarks have only been applied to open pancreatoduodenectomy^10,11^, open distal pancreatectomy^12^ or minimally invasive distal pancreatectomy with splenectomy^13^, and none have been defined for spleen-preserving distal pancreatectomy (SPDP). As previous comparative studies^14–18^ between distal pancreatectomy with and without splenectomy have reported significant differences in intraoperative and postoperative outcomes, separate benchmarks are required for the two procedures.

Interestingly, the approach for defining benchmarks in surgery differs among published studies. The majority of the studies followed the best-patient-in-best-centre methodology, firstly described by Staiger *et al.*^2^, in which benchmarks are derived in a predefined low-risk population as the 75th percentile of the median proportion of outcomes across high-volume units. These benchmarks are supposed to mirror a realistic cut-off value, because not only the top few, but 75 per cent of that median proportion, achieved in high-volume centres, represent the benchmark. However, for the same reason, they may not be considered as intuitive or very strict.

On the contrary, fewer studies have followed the Achievable Benchmark of Care (ABC^TM^; University of Alabama, Birmingham, Alabama, USA) methodology^19^. This aims to present benchmarks as the best achievable outcomes derived from top performers in an unselected population from centres with different volumes. They could be considered too ambitious, but ABC benchmarks do not imply that this outcome always has or can be achieved, but they illustrate the gap between benchmark and personal performance to encourage potential improvement knowing that that the target level has been achieved^19^. Furthermore, these benchmarks are applicable to real-life surgical patients instead of only low-risk patients. To date, it remains unknown whether these two methodologies give similar results or not, and there is no clear consensus on the best benchmark methodology to apply.

This study aimed to establish benchmarks for laparoscopic and robotic SPDP, integrating the ABC and best-patient-in-best-centre methodologies, and to investigate the impact of different methodologies in defining benchmarks and subsequent interpretation and guidance.

## Methods

### Study population and design

Data from patients undergoing either laparoscopically or robotically assisted SPDP for benign and premalignant lesions were extracted from a retrospective database of centres participating in the European Consortium on Minimally Invasive Pancreatic Surgery (E-MIPS) (2006–2019). The anonymous data were collected from the principal investigators of each centre using a Microsoft^®^ Excel^®^ (Microsoft, Redmond, Washington, USA) datasheet. All data were stored in the database and secured with a password. Consecutive patients, aged 18 years or above, were included. Patients who underwent intraoperative splenectomy but were intended for a spleen-preserving procedure were also included. Patients were excluded from a benchmark calculation if there were any missing data for that specific outcome.

### Ethics

The study was conducted according to the principles of the Declaration of Helsinki (64th Fortaleza Brazil, October 2013), and in accordance with the Medical Research Involving Human Subjects Act and STROBE guidelines on reporting of observational studies^20^. The ethical board of Amsterdam UMC waived the need for informed consent owing to the retrospective design.

### Variables and definitions

Preoperative variables included baseline characteristics, such as age, sex, American Society of Anaesthesiologists (ASA) fitness grade^21^, body mass index (BMI), previous abdominal surgery, and tumour size. Indicators of surgical performance were identified based on literature^11,22,23^ and clinical relevance. Ten clinically relevant intraoperative and postoperative outcomes were selected for benchmarking. These included surrogate outcomes of both overall surgical quality (namely duration of operation, intraoperative blood loss, conversion, overall morbidity, major morbidity, duration of hospital stay, readmission, 90-day mortality) and procedure-specific quality (such as failure to preserve the spleen and postoperative pancreatic fistula (POPF)). Postoperative outcomes were recorded up to 90 days after surgery.

Conversion was defined as any procedure that started as minimally invasive but underwent unplanned or unintended laparotomy, or required hand assistance^24^. Overall morbidity included any postoperative complication according to the Clavien–Dindo classification; major morbidity was defined as that with a Clavien–Dindo grade of III or higher^25^. Clinically relevant grade B/C POPF was defined in accordance with the International Study Group of Pancreatic Surgery^26^. Spleen-preserving procedures were classified according to the Kimura^27^ or Warshaw^28^ method. Failure to preserve the spleen included patients in whom spleen preservation was intended before surgery, but intraoperative splenectomy was performed.

### Statistical analysis

Categorical data are presented as proportions, normally distributed continuous data as mean values, and continuous data with a skewed distributed as median (i.q.r.). Normality of distribution was checked by the Kolmogorov–Smirnov test. Mann–Whitney *U*, χ^2^ and Fisher’s exact test were used as appropriate to compare baseline characteristics. Statistical significance was set at two-sided *P* < 0.050. Data were analysed using SPSS^®^ for Windows^®^ version 26.0 (IBM, Armonk, NY, USA).

### Benchmark calculations

#### Best achievable outcome benchmarks (Achievable Benchmark of Care)

The benchmark calculation on the total unselected cohort was performed according to ABC methodology^19^. With this method, benchmark values represent the best achievable outcomes, calculated for the consecutive best performing centres for a specific outcome until at least 10 per cent of the patient pool across all centres is reached. A threshold of 10 per cent is used to ensure that best practice will be measured reliably based on a few remarkable centres, and thus avoiding inclusion of outcomes of average care, usually performed by the majority. Exclusion of centres that provide fewer procedures is not necessary as the calculation adjusts for the impact of procedures in a centre with a small sample size (adjusted performance fraction) without eliminating them.

First, the adjusted performance fraction was calculated by adding 1 to the number of events (numerator) and 2 to the number of patients (denominator), and then dividing the adjusted numerator by the denominator.

Second, the adjusted performance fractions for all the centres were sorted from the lowest (best performing centre) to the highest value. Centres included in the benchmark calculation were the centres with the consecutive lowest adjusted performance fraction until the sum of patients reached at least 10 per cent of the cohort for that specific outcome. The ABC for that outcome was calculated by dividing the sum of all events in the benchmark centres (numerator) by the sum of patients in the benchmark centres (denominator). For this purpose, only centres with at least one event in overall morbidity, major morbidity, conversions, and POPF were included in the analysis as for these outcomes an event rate of zero was not considered achievable in real-life practice. The corresponding 25th, 50th, and 75th percentiles were also reported for each outcome. For continuous outcomes, such as duration of hospital stay and operating time, ABCs were calculated as the 10th percentile of the median value across all centres.

#### Best-patient-in-best-centre method

The benchmark calculation for the best-patient-in-best-centre method was performed as described by Staiger *et al.*^2^. In this methodology, benchmarks are calculated in a predefined low-risk patient cohort treated in expert centres. The benchmarks are represented as the 75th percentile of the medians for each centre for each outcome and considered as a cut-off value, not as best achievable results. These benchmarks reflect realistic and acceptable cut-off values that a performer is at least expected to achieve. Thus, individual values for performers below the benchmark value (75th percentile) indicate acceptable outcomes, whereas values above the benchmark indicate ‘bad or worse’ performance and may require closer attention and evaluation of the potential cause. Centres included in the best-patient-in-best-centre benchmark analysis were required to perform at least 10 minimally invasive distal pancreatectomies annually over the years that they provided patient data. Selection criteria for low-risk patients were determined using those applied in the benchmark analysis for pancreatoduodenectomy^11^, whereas only the surgical criteria were adjusted related to distal pancreatectomy (*Table 1*).

**Table 1:** Selection criteria for low-risk patients included in the best-patient-in-best-centre benchmark analysis

**Inclusion criteria**
Age ≥18 years
Minimally invasive spleen-preserving distal pancreatectomy for benign and premalignant lesions
**Exclusion criteria: surgical**
Extended distal pancreatectomy or multivisceral resection
Previous major abdominal surgery (bariatric and liver surgery)
**Exclusion criteria: co-morbidities**
ASA fitness grade ≥ III
BMI ≥ 35 kg/m²
Cardiac disease, defined as:
CHF onset or exacerbation in the 30 days before surgery
History of angina pectoris within 1 month of surgery
Myocardial infarct within 6 months of surgery
History of percutaneous coronary intervention or cardiac surgery
Atrial fibrillation
Chronic renal failure (MDRD ≥ stage 3), defined as:
GFR < 60 ml per min per 1.73 m2, or serum creatinine >1.8 mg/dl or 160 μmol/l
Chronic obstructive pulmonary disease with FEV1 < 80%
**Exclusion criteria: medication**
Use of one of the following anticoagulants:
Non-vitamin K antagonist oral anticoagulants
Vitamin K antagonist
Clopidogrel
≥ 2 oral antidiabetic drugs or insulin

ASA, American Society of Anaesthesiologists; BMI, body mass index; CHF, congestive heart failure; MDRD, Modification of Diet in Renal Disease; GFR, glomerular filtration rate; FEV1, forced expiratory volume in 1 second.

## Results

### Unselected population

In the study interval, 1230 patients were scheduled for minimally invasive SPDP from 32 centres of the European Consortium on Minimally Invasive Pancreatic Surgery (*Fig. S1*); 1006 patients ultimately underwent minimally invasive SPDP and 224 eventually had an unplanned splenectomy. Among the 1230 patients, 951 (77.3 per cent) underwent laparoscopically assisted and 279 (22.7 per cent) robotically assisted SPDP. Overall, 764 of the patients were women (62.1 per cent), median age was 59 years (i.q.r. 45–68) years, and median BMI was 25.0 (22.3–28.3) kg/m^2^ (*Table 2*). The majority had ASA I–II status (80.4 per cent).

**Table 2:** Baseline characteristics, and perioperative and postoperative outcomes for the total cohort used in the Achievable Benchmark of Care analysis

	LSPDP (*n* = 951)	RSPDP (*n* = 279)	P*
**Baseline characteristics**			
Age (years), median (i.q.r.)	59 (46–69)	57 (44–66)	0.034†
Women	589 (61.9)	175 (62.7)	0.811
ASA fitness grade I–II	576 (80.9)	220 (79.4)	0.599
BMI (kg/m^2^), median (i.q.r.)	25.2 (22.3–28.3)	24.7 (22.1–28.0)	0.208†
Previous abdominal surgery	333 (36.0)	114 (40.9)	0.141
Tumour size > 5 cm	87 (11.3)	24 (11.2)	0.963
**Perioperative and postoperative outcomes**			
Method of spleen preservation			<0.001
Warshaw	235 (33.0)	19 (9.7)	
Kimura	477 (67.0)	176 (90.3)	
Duration of operation (min), median (i.q.r.)	195 (150–254)	262.5 (210–340)	<0.001†
Blood loss (ml), median (i.q.r.)	100 (50–250)	100 (90–200)	0.590†
Conversion	82 (8.6)	19 (6.8)	0.337
Failure to preserve spleen	180 (18.9)	44 (15.8)	0.230
Overall morbidity	469 (49.4)	153 (54.8)	0.108
Major morbidity	138 (14.6)	32 (11.5)	0.187
CR-POPF	164 (17.3)	60 (21.5)	0.108
Duration of hospital stay (days), median (i.q.r.)	7 (5–10)	8 (6–11)	<0.001†
Readmission within 90 days	129 (13.8)	31 (11.2)	0.264
90-day mortality	6 (0.8)	1 (0.4)	0.465

Values are *n* (%) unless otherwise indicated. Percentages may not add up owing to rounding and missing data. ASA, American Society of Anaesthesiologists; BMI, body mass index; LSPDP, laparoscopically assisted spleen-preserving distal pancreatectomy; RSPDP, robotically assisted spleen-preserving distal pancreatectomy; CR-POPF, clinically relevant postoperative pancreatic fistula. *χ^2^ or Fisher’s exact test, except †Mann–Whitney *U* test.

The overall rates of conversion, overall morbidity, and major morbidity were 8.2 per cent (101 patients), 50.6 per cent (622), and 13.9 per cent (170) respectively, with no significant differences between the laparoscopic and robotic approach. The median duration of operation was significantly longer in the robotically assisted SPDP group than in the laparoscopically assisted group (262.5 (210–340) *versus* 195 (150–254) min; *P* < 0.001), as was the median duration of hospital stay (8 (6–11) *versus* 7 (5–10) days; *P* < 0.001). The overall rate of failure to preserve the spleen was 18.2 per cent (224 patients), POPF was reported in 224 patients (18.2 per cent), 160 (13.2 per cent) were readmitted, and 7 died within 90 days of surgery (0.7 per cent), with no significant difference between groups (*Table 2*). A significantly higher proportion of Kimura procedures was performed in the robotically assisted group than the laparoscopic group (90.3 *versus* 67.0 per cent; *P* < 0.001) (*Tables S1 and S2*).

### ABC benchmarks

The best achievable outcome benchmarks for the unselected cohort with their percentile ranks for 10 clinically relevant intraoperative and postoperative domains are reported in *Table 3*. The ABCs for laparoscopically assisted SPDPs among centres were 150 min for duration of operation, 55 ml for intraoperative blood loss, 3.5 per cent for conversion, 0 per cent for failure to preserve the spleen, 27.3 per cent for overall morbidity, 5.1 per cent for major morbidity, 3.6 per cent for POPF, 5 days for duration of hospital stay, 2.9 per cent for readmission, and 0 per cent for 90-day mortality.

**Table 3:** Achievable Benchmark of Care benchmarks and corresponding percentiles of laparoscopically and robotically assisted spleen-preserving distal pancreatectomy

	ABC best achievable outcome	25th percentile	50th percentile	75th percentile
**Duration of operation (min)**				
LSPDP	150	180	220	255
RSPDP	207	223	263	324
**Intraoperative blood loss (ml)**				
LSPDP	55	92.5	100	218.8
RSPDP	100	100	125	175
**Conversion (%)**				
LSPDP	3.5	4.8	11.1	16.9
RSPDP	1.7	6.8	10.1	12.4
**Failure to preserve spleen (%)**				
LSPDP	0	4	16.7	29.5
RSPDP	1.7	0	21.4	31.3
**Overall morbidity (%)**				
LSPDP	27.3	40.0	53.1	66.7
RSPDP	34	46.8	57.8	70.3
**Major morbidity (%)**				
LSPDP	5.1	6.3	12.1	20.5
RSPDP	3.3	6.8	10.1	20
**CR-POPF (%)**				
LSPDP	3.6	9.4	17.3	26.6
RSPDP	7.1	13.3	20	30.3
**Duration of hospital stay (days)**				
LSPDP	5	6	7	8
RSPDP	6	7	8	11
**Readmission (%)**				
LSPDP	2.9	6.7	15.2	22.7
RSPDP	5.4	0	6.7	17.6
**90-day mortality (%)**				
LSPDP	0	0	0	0
RSPDP	0	0	0	0

The analysis included 951 patients who underwent laparoscopically assisted (LSPDP) and 279 who underwent robotically assisted (RSPDP) spleen-preserving distal pancreatectomy. ABC, Achievable Benchmark of Care; CR-POPF, clinically relevant postoperative pancreatic fistula.

The ABCs for robotically assisted SPDP among centres were 207 min for duration of operation, 100 ml for intraoperative blood loss, 1.7 per cent for conversion, 1.7 per cent for failure to preserve the spleen, 34 per cent for overall morbidity, 3.3 per cent for major morbidity, 7.1 per cent for POPF, 6 days for duration of hospital stay, 5.4 per cent for readmission, and 0 per cent for 90-day mortality (*Table 3*).

### Best-patient-in-best-centre benchmarks

From the total cohort of 1230 patients treated in 32 centres, 764 (62 per cent) low-risk patients treated in 23 centres were identified for the best-patient-in-best-centre benchmark analysis. Exclusion and inclusion criteria for the low-risk cohort are reported in *Table 1*. Of the 764 procedures, 602 (79 per cent) were laparoscopically assisted and 162 (21 per cent) were robotically assisted. Patient and operative characteristics are summarized in *Table 4*. The benchmarks cut-offs for laparoscopically assisted SPDP were 254 min for duration of operation, 150 ml for intraoperative blood loss, 5.8 per cent for conversion, 29.9 per cent for failure to preserve the spleen, 62.7 per cent for overall morbidity, 20.4 per cent for major morbidity, 23.8 per cent for POPF, 8 days for duration of hospital stay, 20 per cent for readmission, and 0 per cent for 90-day mortality (*Table 5*).

**Table 4:** Baseline characteristics, and perioperative and postoperative outcomes for the low-risk cohort used in the best-patient-in-best-centre benchmark analysis

	LSPDP (*n* = 602)	RSPDP (*n* = 162)	P*
**Baseline characteristics**			
Age (years), median (i.q.r.)	56 (42–66)	52 (41–65)	0.039†
Women	409 (67.9)	103 (63.6)	0.295
ASA fitness grade I–II	434 (100)	161 (100)	–
BMI (kg/m^2^), median (i.q.r.)	24.8 (22.0–27.5)	24.2 (22.1–26.8)	0.723†
Previous abdominal surgery	201 (33.9)	57 (35.2)	0.759
Tumour size > 5 cm	61 (12.4)	21 (16.5)	0.217
**Perioperative and postoperative outcomes**			
Method of spleen preservation			<0.001
Warshaw	157 (33.2)	11 (9.2)	
Kimura	316 (66.8)	108 (90.8)	
Duration of operation (min), median (i.q.r.)	189 (140–241)	240 (195–336)	<0.001†
Blood loss (ml), median (i.q.r.)	100 (50–200)	100 (80–200)	0.620†
Conversion	40 (6.7)	10 (6.2)	0.826
Failure to preserve spleen	105 (17.4)	25 (15.4)	0.546
Overall morbidity	297 (49.3)	78 (48.1)	0.788
Major morbidity	87 (14.5)	14 (8.6)	0.052
CR-POPF	101 (16.8)	29 (17.9)	0.742
Duration of hospital stay (days), median (i.q.r.)	7 (6–10)	8 (6–10)	0.010†
Readmission within 90 days	83 (13.8)	18 (11.1)	0.368
90-day mortality	2 (0.5)	0 (0)	0.387

Values are *n* (%) unless otherwise indicated. Percentages may not add up owing to rounding and missing data. ASA, American Society of Anaesthesiologists; BMI, body mass index; LSPDP, laparoscopically assisted spleen-preserving distal pancreatectomy; RSPDP, robotically assisted spleen-preserving distal pancreatectomy; CR-POPF, clinically relevant postoperative pancreatic fistula. *χ^2^ or Fisher’s exact test, except †Mann–Whitney *U* test.

**Table 5:** Best-patient-in-best-centre 75th percentiles (benchmark cut-offs) and 25th percentiles in comparison to those for Achievable Benchmark of Care best achievable benchmarks and percentiles for laparoscopically assisted spleen-preserving distal pancreatectomy

	Low-risk patients undergoing LSPDP (*n* = 602)		All patients undergoing LSPDP (*n* = 951)			
	BPBC 25th percentile	BPBC 75th percentile (benchmark cut-off)	ABC best achievable outcome	ABC 25th percentile	ABC 50th percentile	ABC 75th percentile
Duration of operation (min)	180	254	150	180	220	255
Intraoperative blood loss (ml)	65	150	55	92.5	100	218.8
Conversion (%)	0	5.8	3.5	4.8	11.1	16.9
Failure to preserve spleen (%)	4.7	29.9	0	4.0	16.7	29.5
Overall morbidity (%)	37.2	62.7	27.3	40.0	53.1	66.7
Major morbidity (%)	5.6	20.4	5.1	6.3	12.1	20.5
CR-POPF (%)	0.6	23.8	3.6	9.4	17.3	26.6
Duration of hospital stay (days)	6	8	5	6	7	8
Readmission (%)	8.1	20	2.9	6.7	15.2	22.7
90-day mortality (%)	0	0	0	0	0	0

LSPDP, laparoscopically assisted spleen-preserving distal pancreatectomy; BPBC, best patient in best centre; ABC, Achievable Benchmark of Care; CR-POPF, clinically relevant postoperative pancreatic fistula.

Robotically assisted SPDP benchmark cut-offs were 262.5 for duration of operation, 195 ml for intraoperative blood loss, 8.2 per cent for conversion, 27.3 per cent for failure to preserve the spleen, 55.7 per cent for overall morbidity, 14 per cent for major morbidity, 24.2 per cent for POPF, 8 days for duration of hospital stay, 15.1 per cent for readmission, and 0 per cent for 90-day mortality (*Table 6*).

**Table 6:** Best-patient-in-best-centre 75th percentiles (benchmark cut-offs) and 25th percentiles in comparison to those for Achievable Benchmark of Care best achievable benchmarks and percentiles for robotically assisted spleen-preserving distal pancreatectomy

	Low-risk patients undergoing RSPDP (*n* = 162)		All patients undergoing RSPDP (*n* = 279)			
	BPBC 25th percentile	BPBC 75th percentile (benchmark cut-off)	ABC best achievable outcome	ABC 25th percentile	ABC 50th percentile	ABC 75th percentile
Duration of operation (min)	211.4	262.5	207	223	263	324
Intraoperative blood loss (ml)	100	195	100	100	125	175
Conversion (%)	0	8.2	1.7	6.8	10.1	12.4
Failure to preserve spleen (%)	1	27.3	1.7	0	21.4	31.3
Overall morbidity (%)	41	55.7	34	46.8	57.8	70.3
Major morbidity (%)	4.6	14	3.3	6.8	10.1	20
CR-POPF (%)	0	24.2	7.1	13.3	20	30.3
Duration of hospital stay (days)	6	8	6	7	8	11
Readmission (%)	7.7	15.1	5.4	0	6.7	17.6
90-day mortality (%)	0	0	0	0	0	0

RSPDP, robotically assisted spleen-preserving distal pancreatectomy; BPBC, best patient in best centre; ABC, Achievable Benchmark of Care; CR-POPF, clinically relevant postoperative pancreatic fistula.

## Discussion

This pan-European multicentre retrospective study identified benchmarks for 10 clinically relevant surgical outcomes after laparoscopically and robotically assisted SPDP using the 2 most widely established and validated methodologies^2,19^, applied to unselected and low-risk patients.

Based on an unselected population of 951 laparoscopically and 279 robotically assisted procedures from 32 European centres, the ABC best achievable values for both procedures were quite comparable for most parameters. The biggest differences were found in duration of surgery and POPF rates, favouring the laparoscopic approach. The superiority of the laparoscopic approach in terms of operating time has been noted in previous cohort studies and meta-analyses^29–31^. According to a recently published fistula risk score for distal pancreatectomy, longer operating times can increase the risk of POPF^32^, which might explain the higher POPF rate after robotically assisted SPDP. None of the other variables included in the new fistula risk score differed significantly between the two groups. For both procedures, conversion rates were low: 3.5 per cent for laparoscopically assisted and 1.7 per cent for robotically assisted SPDP. In recent studies^15,18,33^, conversion rates have varied from 0 to 9 per cent, making the ABC values of 3.5 and 1.7 per cent obtained here seem realistic. The lower ABC conversion rate for the robotic procedure aligns with the results of a recent meta-analysis^31^ that reported lower conversion rates for robotically compared with laparoscopically assisted SPDP. This could be attributed to the features of the robotic system, allowing greater dexterity and three-dimensional vision.

A remarkable outcome of this study is the ABC rate of 0 per cent for laparoscopically assisted and 1.7 per cent for robotically assisted SPDP for failure to preserve the spleen. Centres with no events were included in the benchmark calculation, as an event rate of zero was considered feasible and what should be strived for, even in real-life practice. The current literature confirms the feasibility of such ambitious values, as 100 per cent rates of successful spleen preservation have been reported in previous studies^33–35^. Although other studies^29–31^ have pointed towards the superiority of the robotic approach in terms of splenic preservation, the present findings do not confirm this.

Profound differences were noted between the ABC benchmarks for the unselected cohort and the best-patient-in-best-centre enchmarks for the low-risk cohort. As the best-patient-in-best-centre methodology aims to provide cut-off values rather than best achievable results, these benchmarks are more lenient than the ABC benchmarks and were more likely comparable to the ABC medians or 75th percentiles. The largest differences between the best-patient-in-best-centre 75th percentiles and the ABC 75th percentiles (that is differences between low-risk cohort and total cohort) were found for conversion, morbidity, POPF, and readmission rates; the best-patient-in-best-centre benchmarks were lower and thus stricter. These findings suggest that a low-risk cohort mainly results in lower rates of conversion, morbidity, and readmission, and to a lesser extent affects parameters such as operating time, duration of hospital stay, and spleen preservation. Previous literature supports these findings given the exclusion criteria used for the low-risk cohort in which the best-patient-in-best-centre benchmarks were established; extended or multivisceral resections, ASA grade at least III, major previous abdominal surgery, and BMI 35 kg/m² or higher have been associated with higher rates of conversion and postoperative morbidity^36–39^. Excluding such patients may have resulted in better outcomes and thus led to stricter best-patient-in-best-centre 75th percentiles compared with the ABC 75th percentiles.

The differences in benchmark outcomes obtained with the two methodologies show that applying the concept of benchmarking necessitates realism in the choice of methodology and that the two approaches imply critically divergent interpretation. The present study has shown that the type of benchmark used in daily practice should depend on two factors. The first of these is the purpose of benchmarking. If the purpose is to compare outcomes between departments or hospitals, it is recommended to use best-patient-in-best-centre benchmark cut-offs to illustrate an accepted level of performance. On the contrary, if the intention is to compare individual surgeons and motivate them towards superior performance, ABC benchmarks, representing the best achievable outcomes, would be more useful. Second, the type of cohort must be considered in the choice of benchmark. As the methodologies have been developed and validated in different patient cohorts, and the benchmark values in the present study have been obtained according to this, the benchmarks should be applied to the appropriate patient cohort to generate reliable and equal comparisons. Discrepancy may arise when ABC benchmarks are applied to a low-risk cohort, as they will most likely be more easily achieved; on the other hand, an unselected population would be expected to perform outside the benchmark cut-offs when best-patient-in-best-centre benchmarks are applied.

The preferred type of benchmark remains debatable, as clarity on the most reliable or appropriate methodology is still lacking. Recently, a standardized methodology for establishing benchmarks based on a Delphi consensus was published^40^, endorsing the best-patient-in-best-centre methodology of the present study. However, even though many points were clarified during this consensus, uncertainties remain on the correct use of benchmarks in clinical practice and their generalizability to non-low-risk patients. The present authors wonder whether it is time for the surgical community to consider a new or modified benchmark method, one that considers both best achievable and acceptable results, balances between ABC and best-patient-in-the-best-centre methods, and can identify personalized benchmarks based on different risk groups. Clinical expertise and judgement on the subject of benchmarking are needed to ensure equal and accurate comparison of clinical outcomes in (pancreatic) surgery.

The results of this study should be interpreted in the light of several limitations. First, owing to the retrospective design and the wide time span of the study, confounding factors and changes in (post)operative policies and definitions of outcomes over time may have influenced outcomes, such duration of hospital stay. In addition, because no data on the learning curve phase at the time of surgery were available, it is feasible that surgeons provided data during different phases of their learning curve. As proficiency learning curves may be quite long, this could have biased the results^41^. Second, significant differences in outcomes such as major complications were found when comparing the Kimura and Warshaw methods, and might have influenced some benchmark outcomes. Third, only 279 robotically assisted procedures were collected in the database, which may be on the low side and therefore could raise doubts about the reliability of benchmarks for robotic procedures compared with the laparoscopic benchmarks. A major strength of this study is that the data were retrieved from a pan-European database, making the results generalizable and better reflecting the real-world scenario.

## Supplementary Material

znac352_Supplementary_Data

## Data Availability

Manuscript data is available on request from the authors.
