# Supplementary material for: Benchmarking of robotic and laparoscopic spleen-preserving distal pancreatectomy by using two different methods
Source: Br J Surg. 2022 Nov 2;110(1):76–83. doi: 10.1093/bjs/znac352 (PMC10364499; doi:10.1093/bjs/znac352)
Supplement: znac352_Supplementary_Data [file znac352_supplementary_data.docx]

**FIGURE S1. Distribution of cases among all centers**

**TABLE S1. Peri – and postoperative outcomes of Kimura and Warshaw spleen-preserving minimally invasive distal pancreatectomy**

|  | MI-Kimura (n=653) | MI-Warshaw (n=254) | *p* |
| --- | --- | --- | --- |
| Operative time, minutes, median, [IQR] | 210 [153 – 268] | 190 [135 – 255] | **0.027** |
| Intraoperative blood loss, mL, median, [IQR] | 100 [50 – 200] | 150 [75 – 300] | **<0.001** |
| Conversion, n, (%) | 38 (5.8) | 9 (3.5) | 0.165 |
| Failure to preserve, n (%) | Not applicable,  no Kimura performed | Not applicable,  no Warshaw performed |  |
| Complications, n, (%) | 298 (45.7) | 144 (56.7) | **0.003** |
| Severe complications, n, (%) | 77 (11.8) | 47 (18.5) | **0.009** |
| CR-POPF, n (%) | 110 (16.9) | 48 (18.9) | 0.476 |
| Length of hospital stay, days, median, [IQR] | 7 [6 – 9] | 8 [6 – 13] | **<0.001** |
| Readmissions, n, (%) | 69 (10.8) | 39 (15.5) | 0.054 |
| 90 day mortality, n (%) | 0 (0) | 3 (2.3) | **<0.001** |

*Values in parentheses are percentages unless mentioned otherwise. Percentages may not add up due to rounding and missing data. MI = minimally invasive, IQR = inter quartile range, CR-POPF = clinically relevant postoperative pancreatic fistula*

**TABLE S2. Peri – and postoperative outcomes of Kimura and Warshaw spleen-preserving robotic and laparoscopic distal pancreatectomy**

|  | LSPDP (n=951) | | *p* | RSPDP (n=279) | | *p* |
| --- | --- | --- | --- | --- | --- | --- |
|  | **Kimura  *(n=477)*** | **Warshaw (n=235)** |  | **Kimura (n=176)** | **Warshaw (n=19)** |  |
| Operative time, minutes, median, [IQR] | 190  [145 – 242] | 180  [130 – 240] | 0.489 | 240 [195 – 326] | 274 [218 – 360] | 0.344 |
| Intraoperative blood loss, mL, median, [IQR] | 100 [50 – 200] | 100  [50 – 300] | **<0.001** | 100  [100 – 200] | 200  [100 – 300] | **0.048** |
| Conversion, n, (%) | 33 (6.9) | 8 (3.4) | 0.058 | 5 (2.8) | 1 (5.3) | 0.561 |
| Failure to preserve, n (%) | Not  applicable,  no Kimura performed | Not applicable,  no Warshaw performed | - | Not applicable,  no Kimura performed | Not applicable,  no Warshaw performed | - |
| Complications, n, (%) | 208 (43.7) | 136 (57.9) | **<0.001** | 90 (51.1) | 8 (42) | 0.454 |
| Severe complications, n, (%) | 59 (12.4) | 44 (18.7) | **0.025** | 18 (10.2) | 3 (15.8) | 0.457 |
| CR-POPF, n (%) | 68 (14.3) | 45 (19.1) | 0.098 | 42 (23.9) | 3 (15.8) | 0.427 |
| Length of hospital stay, days, median, [IQR] | 7  [5 – 9] | 8  [6 – 13] | **<0.001** | 8 [6 – 11] | 8  [5 – 10] | 0.388 |
| Readmissions, n, (%) | 53 (11.4) | 37 (15.8) | 0.105 | 16 (9.1) | 2 (11.1) | 0.778 |
| 90 day mortality, n (%) | 0 (0) | 2 (1.8) | **0.007** | 0 (0) | 1 (5.3) | **0.003** |

*Values in parentheses are percentages unless mentioned otherwise. Percentages may not add up due to rounding and missing data LSPDP = laparoscopic spleen-preserving distal pancreatectomy, RSPDP = robotic spleen-preserving distal pancreatectomy, CR-POPF = clinically relevant postoperative pancreatic fistula*
